# Educational neuromyths in higher education: beliefs about the brain and learning among university faculty members

**DOI:** 10.3389/fpsyg.2026.1952466

**Published:** 2026-09-09

**Authors:** Fernando Pesantez Aviles, Angel Torres-Toukoumidis, Isidro Marín-Gutiérrez, Santiago Vintimilla

**Affiliations:** 1 Salesian Polytechnic University, Cuenca, Ecuador; 2 Universidad de Malaga, Málaga, Spain

**Keywords:** educational neuromyths, evidence-based education, faculty development (FD), higher education, neuroscience literacy, university faculty members

## Abstract

Educational neuromyths are plausible but unsupported generalizations about the brain that can shape instructional decisions. This cross-sectional study examined their endorsement among 269 participating university faculty members in Ecuador. Recruitment followed a non-probability procedure that was not documented in the available records, so the results describe this sample and are not nationally representative. The Spanish-language survey contained 21 belief statements rated on a 5-point Likert scale. Based on published reviews and meta-analyses, 11 statements were classified as established neuromyths or unsupported general claims, four as evidence-aligned statements, and six as context-dependent or overgeneralized statements that were not scored dichotomously. Item agreement was summarized with Wilson 95% confidence intervals. An exploratory 15-item scientific-alignment indicator and a clustered generalized estimating equation model were also used. Agreement was highest for learning-style matching (90.7%), generalized transfer from brain games (83.3%), hemispheric dominance (75.0%), cross-lateral integration exercises (74.7%), and use of only a small part of the brain (72.8%). Participants endorsed a mean of 6.02 of 11 neuromyths, and 98.1% endorsed at least one, while agreement with four evidence-aligned statements averaged 91.9%. The mean scientific-alignment score was 44.0%. In the exploratory adjusted model, women had higher odds of an aligned response than men (adjusted odds ratio = 1.58, 95% confidence interval [1.17, 2.13]), an exploratory association detected under the primary specification, which did not survive false-discovery-rate correction when neutral responses were excluded, and that requires replication and should not be read as an inherent gender difference; no statistically robust association was detected for experience, academic degree, specialized training, or most information sources, although the absence of a detected association does not establish that these factors are unrelated to alignment. The findings indicate that, in this sample, accurate knowledge and neuromyths can coexist at participant level: the correlation between evidence-aligned knowledge and neuromyth endorsement was weak and positive (Spearman *ρ* = 0.25, 95% CI [0.13, 0.36]) rather than negative as a deficit account predicts, supporting targeted refutation and evidence-appraisal training rather than generic exposure to neuroscience facts.

## 1 Introduction

Neuroscience occupies an increasingly conspicuous place in teacher education and professional development, a prominence that proves productive when findings on memory, attention, sleep, stress, and plasticity are construed within the broader science of learning. The difficulty arises when conditional findings are transmuted into universal prescriptions as they circulate through commercial training, popular media, social networks, and simplified digests. Under this dynamic, claims such as matching instruction to a putative learner type or training a supposedly weaker hemisphere acquire a scientific resonance they have not earned, for the prescribed practice is rarely shown to improve learning (Howard-Jones, 2014; Jolles and Jolles, 2021).

A neuromyth, as Dekker et al. (2012) and Howard-Jones (2014) delineate it, constitutes a misconception generated by the misunderstanding or overgeneralization of established findings about the brain. Such claims almost invariably harbor a kernel of truth: the hemispheres do exhibit relative specializations, neural systems are demonstrably plastic, and learners undeniably vary. Yet complex academic tasks recruit interacting networks, and individual variability does not, in itself, warrant the fixed visual, auditory, or kinesthetic categories that matched instruction presupposes. The passage from a laboratory finding to a classroom prescription demands several inferential steps that are seldom made explicit, inasmuch as neural involvement in a task does not specify how that task ought to be taught, and short-term performance change does not evidence durable learning or transfer.

The consequences of neuromyths for educational decision-making operate largely through opportunity costs. Learner labels may constrict instructional opportunities, while unsupported diagnostics, brain-training products, and professional-development packages can displace time and funding from practices with sturdier evidential foundations, among them spacing, retrieval, feedback, adequate sleep, and content-appropriate multimodal representation (Pashler et al., 2008; Simons et al., 2016; Waterhouse, 2023). The learning-style matching hypothesis proves especially seductive because it presents itself as learner-centered, yet it hinges on a crossover interaction whereby learners assigned to different styles benefit differentially from distinct instructional modes. Pashler et al. (2008) found the evidence for this standard inadequate, and a subsequent meta-analysis by Clinton-Lisell and Litzinger (2024) reported only a small pooled advantage accompanied by limited crossover evidence and considerable methodological heterogeneity. Multiple-intelligences labels, cross-lateral programs, and brain-training claims extend genuine phenomena beyond their warranted scope in comparable fashion: improvement on a practiced task is not tantamount to broad cognitive gain, and successive reviews have declined to corroborate the sweeping benefits routinely advertised for commercial brain training (Ophir et al., 2009; Simons et al., 2016).

Substantial endorsement has been documented among teachers across several national contexts, including the United Kingdom, Netherlands, Spain, and Latin America (Dekker et al., 2012; Ferrero et al., 2016; Gleichgerrcht et al., 2015). Education or neuroscience training tends to attenuate, though not to eliminate, these beliefs (Macdonald et al., 2017). This consideration acquires particular weight in higher education, where disciplinary expertise does not necessarily encompass preparation in the science of learning. Crucially, accurate knowledge and misconception may coexist rather than exclude one another: Grospietsch and Mayer (2019) demonstrate that pre-service teachers can recognize valid principles of learning and memory while simultaneously endorsing specific neuromyths. Recent evidence reinforces this coexistence. von Hausen et al. (2026), in a cross-domain study of Chilean special-school educators published in this journal, establish that neuroscience literacy does not function as a single, domain-general safeguard against neuromyth endorsement, its protective association varying instead with the particular claim under evaluation; Novak-Geiger (2023) likewise reports persistent misconceptions among psychology students only marginally distinct from those of pre-service teachers. It follows that appending general neuroscience facts may prove less efficacious than naming the misconception, explicating its intuitive appeal and unsupported inference, and furnishing a practicable evidence-based alternative (Rousseau, 2021), all the more so because the magnitude and durability of corrections are contingent on message design, repetition, source, and prior commitment (Chan and Albarracín, 2023).

Not every neuroscience-related survey statement admits a simple true-or-false verdict. Claims concerning chronotype, hydration, supplementation, music, sex differences, or instructional methods are conditioned by population, dose, outcome, and wording, and each compresses heterogeneous evidence into phrasing too broad to sustain a defensible binary score (Adan et al., 2012; Hyde, 2005; Masento et al., 2014). Latin American studies evidence considerable endorsement and thereby justify context-specific analyses (Gleichgerrcht et al., 2015; von Hausen et al., 2026); national generalization, however, requires a documented sampling frame and recruitment procedure that the present data do not afford.

In light of the foregoing, this study reanalyzed item-level responses from university teachers in Ecuador, separating established neuromyths or unsupported general claims from evidence-aligned and context-dependent statements. Its general objective was to characterize the endorsement of educational neuromyths and its coexistence with evidence-aligned knowledge among university teachers in Ecuador, and to examine whether scientifically aligned responses are conditioned by demographic and professional factors. Three specific objectives operationalized this aim: (1) to quantify, at the item level and with interval estimation, the prevalence of established educational neuromyths; (2) to determine the extent to which accurate knowledge coexists with neuromyth endorsement, thereby adjudicating between a deficit model and a coexistence model; and (3) to examine whether scientifically aligned responses are associated with gender, age, teaching experience, academic degree, education-related training, or reported information sources.

Consonant with these objectives and with the literature reviewed above, four hypotheses were advanced. H1 (ubiquity) posited that established educational neuromyths would be prevalent, such that most participants would endorse several simultaneously. H2 (coexistence) posited that accurate knowledge and neuromyth endorsement would co-occur rather than exclude one another, so that high adherence to evidence-aligned principles would coincide with substantial neuromyth endorsement, contravening a simple deficit model. H3 (weak categorical prediction) posited that broad categories of academic degree, teaching experience, and professional training would not robustly predict scientifically aligned responding, in keeping with prior evidence that factual knowledge does not reliably immunize educators against misconceptions (Macdonald et al., 2017; von Hausen et al., 2026). Finally, an exploratory hypothesis, H4, anticipated that at least one demographic or source-related covariate would be associated with alignment; because the present work is a secondary reanalysis rather than a preregistered design, H4 is treated as exploratory and hypothesis-generating rather than confirmatory. The design is cross-sectional and consequently cannot establish causation or temporal persistence.

## 2 Literature review

### 2.1 From neuroscientific findings to educational Neuromyths

The transit from a laboratory observation to a classroom prescription traverses several inferential steps that are seldom rendered explicit. That a task recruits a given neural system does not, of itself, specify how the task ought to be taught, and a short-term change in performance furnishes no warrant for durable learning or transfer; technical vocabulary can nonetheless lend an unearned authority to instructional claims that have never been tested (Howard-Jones, 2014). The persistence of the notion that people use only a small fraction of the brain, together with the strong-form left-brain and right-brain dichotomy, illustrates the pattern with particular clarity: cerebral systems do exhibit relative specialization, yet complex learning mobilizes distributed and interacting networks, so that selective activation at a given instant constitutes no evidence that the remainder lies dormant. Such claims endure precisely because they are memorable and appear to explain the everyday differences teachers observe (Howard-Jones, 2014; Torrijos-Muelas et al., 2021).

Learning-style matching commands assent because it presents itself as learner-centered, though its empirical standard is exacting, requiring a crossover interaction in which learners assigned to distinct styles derive differential benefit from distinct instructional modes. Pashler et al. (2008) judged the evidence for this standard inadequate, and a subsequent meta-analysis reported only a modest pooled advantage attended by scant crossover evidence and considerable methodological heterogeneity (Clinton-Lisell and Litzinger, 2024). Multiple representations may well assist comprehension when the content warrants them; fixed visual, auditory, or kinesthetic labels, by contrast, ought not to govern institutional policy. Cognate overextensions recur in multiple-intelligences taxonomies and cross-lateral programs, which convert broad intuitions about ability or coordination into unsupported prescriptions for individualized instruction (Waterhouse, 2023), and in brain-game claims, which fail the same transfer test, inasmuch as improvement on a practiced puzzle is not equivalent to broad cognitive gain and successive reviews decline to corroborate the sweeping benefits advertised for commercial training (Ophir et al., 2009; Simons et al., 2016).

### 2.2 Accurate knowledge and misconception in coexistence

A deficit model construes neuromyth endorsement as the mere absence of facts, yet the evidence resists so parsimonious a reading, for heightened interest or additional training may augment factual knowledge even as familiar myths persist undisturbed (Grospietsch and Mayer, 2019; Macdonald et al., 2017). Accurate and inaccurate representations are accordingly better examined item by item than arrayed along a single continuum. Confidence and accumulated experience can, moreover, entrench an existing explanation: a teacher who observes that a student favors diagrams may infer that matched visual instruction produced the learning, when prior knowledge, motivation, task design, or the general advantage of combining words with images offer equally plausible accounts (Rollwage et al., 2020).

Corrections are, on balance, effective, but their magnitude and durability depend upon message design, repetition, source credibility, and prior commitment (Chan and Albarracín, 2023). It follows that appending general neuroscience facts is apt to prove less efficacious than naming the misconception, exposing its intuitive appeal and its unsupported inference, and furnishing a practicable evidence-based alternative (Rousseau, 2021). This coexistence, rather than any simple deficit, has lately been corroborated across contexts: von Hausen et al. (2026) establish that neuroscience literacy does not operate as a single, domain-general safeguard, its protective association varying instead with the particular claim under evaluation, while Novak-Geiger (2023) documents persistent misconceptions among psychology students only marginally distinct from those of pre-service teachers.

### 2.3 Professional learning and the appraisal of evidence

Direct refutation, explanatory feedback, and structured opportunities to apply corrected concepts can diminish neuromyth endorsement, though the effects are at times incomplete or transient (Rousseau, 2021). A course in the science of learning can improve both the rejection of myths and neuroscience literacy when evidence is explicitly bound to educational reasoning (Ferreira and Rodríguez, 2022). Of comparable importance is the appraisal of sources: educators are well served by asking whether a claim rests upon an educational outcome, a neural correlate, an expert opinion, or a commercial testimonial, and by interrogating the population, comparison, outcome, duration, and transfer that the claim presupposes. Evidence concerning dehydration or chronotype, to take two instances, does not of itself license universal prescriptions for greater fluid intake or individualized scheduling (Adan et al., 2012; Masento et al., 2014).

Latin American studies, finally, document substantial endorsement and thereby justify context-specific analysis (Gleichgerrcht et al., 2015). Such work can identify locally salient claims and orient professional development toward them, though national generalization remains contingent upon a documented sampling frame and recruitment procedure, a caveat that circumscribes the reach of the present data and motivates the item-level, interval-estimated approach adopted below.

## 3 Materials and methods

### 3.1 Design and participants

This study used a quantitative, cross-sectional survey design. The available dataset contained 269 records from university faculty members in Ecuador, all of whom selected the consent response. Data collection was reported to have occurred between November 2025 and February 2026. The analysis describes the participating sample and tests associations within it; it does not claim national representativeness. Table 1 summarizes the characteristics of the participating sample. The dataset was made available to the present authors for secondary analysis. The records documenting recruitment—the number and type of participating institutions, their geographical distribution, the recruitment channels and invitation dates, the eligibility and exclusion criteria, the number of faculty invited, the number responding, the number excluded, and the resulting response rate—could not be recovered and are therefore unavailable. No sampling frame was constructed and participation was self-selected, so the sample is a non-probability convenience sample and no response rate can be reported. No prospective sample-size calculation was performed. For descriptive purposes, *N* = 269 yields a Wilson 95% confidence interval no wider than ±5.9 percentage points around a proportion of 0.50, and appreciably narrower intervals for the more extreme proportions reported below; this precision is adequate for item-level estimation within the participating sample but does not license inference to the national population of Ecuadorian university faculty.

**Table 1:** Participant characteristics (*N* = 269).

Characteristic	Category	*n*	%
Gender	Women	131	48.7
	Men	138	51.3
Age	18–29 years	15	5.6
	30–44 years	130	48.3
	45–59 years	101	37.5
	60 years or older	23	8.6
Teaching experience	0–5 years	98	36.4
	6–10 years	40	14.9
	11–20 years	73	27.1
	21 years or more	58	21.6
Highest academic degree	Technical/technological	1	0.4
	Bachelor/engineering	5	1.9
	Specialization	23	8.6
	Master’s	188	69.9
	Doctorate	52	19.3
Education-related training	None	46	17.1
	Short pedagogical courses	138	51.3
	Bachelor’s in education/pedagogy	13	4.8
	Specialization in education/pedagogy	17	6.3
	Master’s/doctorate in education or allied field	55	20.4
Information source*	Formal academic curriculum	75	27.9
	Courses or professional development	165	61.3
	Scientific books or journals	117	43.5
	Social media	60	22.3
	Artificial intelligence tools	70	26.0
	None	16	5.9

*Multiple responses were permitted for information sources; percentages therefore do not sum to 100%.

The participant profile reported here was verified at this stage of revision against the source records held by the original data-collection team. That verification confirmed the figures reported in the first version of this manuscript: the 269 consented respondents comprise 131 women (48.7%) and 138 men (51.3%). In the course of it, the gender variable of 24 records was corrected against the source responses, the raw survey export having recorded them inaccurately; no other variable and no other record was modified, and the analytical *N* remains 269. The corrected file is openly deposited and identified in the Data Availability Statement, and it is the definitive analytical dataset for every result, table and figure reported in this article. All participant-level estimates were re-estimated on it, as set out in the Data Analysis subsection below.

### 3.2 Instrument and item classification

The workbook contained 28 columns: one consent item, six background or source questions, and 21 neuroscience-and-learning statements. Background variables covered gender, age, teaching experience, highest academic degree, education-related training, and sources of information about neuroscience and learning. Respondents rated each belief statement on a five-point scale from 1 (strongly disagree) to 5 (strongly agree).

For this reanalysis, the 21 statements were classified by comparing their wording with peer-reviewed reviews, meta-analyses, and primary research indexed in major citation databases. Eleven were designated established neuromyths or unsupported general claims because they asserted broad educational or cognitive effects not supported as general rules (e.g., Lehtonen et al., 2018). Four were designated evidence-aligned statements: lifelong plasticity, adequate sleep and memory consolidation, spaced practice, and the potential effect of prolonged stress on learning (Cepeda et al., 2006; Magee and Grienberger, 2020; McEwen and Morrison, 2013; Rasch and Born, 2013). Six were designated context-dependent or overgeneralized and excluded from the scientific-alignment indicator; they concerned project-based learning, temporary effects of classical music, supplementation in healthy students, elevated hydration, chronotype-based scheduling, and average sex differences. Each of these compresses heterogeneous evidence into wording too broad for a defensible binary score; for instance, preventing dehydration can matter without implying that greater intake always improves cognition, and average group differences do not justify deterministic instructional grouping (Hyde, 2005; Masento et al., 2014). This decision was made to avoid forcing qualified claims into a binary true/false framework. The classification was established on evidentiary grounds before the item response distributions were inspected and was not revised thereafter; it is described as *a priori* only in this restricted sense, and no preregistration was filed. The classification was carried out by the research team and was not independently double-coded, so no inter-rater agreement statistic is available; this is stated as a limitation below. The complete original Spanish wording and its English translation, the assigned category, the direction of response counted as aligned, the evidentiary rationale, and the key supporting source or sources are reported, statement by statement, for all 21 items in Supplementary Table S1, appended to this article.

### 3.3 Data analysis

Responses were cleaned by standardizing whitespace in category labels and mapping the Likert options from 1 to 5. For each statement, agreement combined agree and strongly agree, disagreement combined disagree and strongly disagree, and the middle category was retained as neutral. Agreement proportions are reported with Wilson 95% confidence intervals. One participant had a missing response to Item 8.1 and one missing response to Item 8.2; all other belief items were complete.

An exploratory scientific-alignment indicator was calculated from 15 unambiguous statements. For the 11 neuromyth or unsupported statements, disagreement counted as aligned; for the four evidence-aligned statements, agreement counted as aligned. Neutral responses and responses on the scientifically unsupported side counted as not aligned. Each participant’s score was the percentage of available aligned responses. A separate count summarized how many of the 11 neuromyth statements each participant endorsed. Three sensitivity analyses were added at revision: (a) neutral responses excluded from the binary outcome and treated as missing; (b) neutral responses modeled as a separate category; and (c) an ordinal cumulative-logit model retaining all five Likert categories. The overall alignment estimate and the gender association are reported under each specification because the instrument was not originally validated as a unidimensional scale, item-level prevalence was treated as the primary outcome and the composite as an exploratory index of response alignment rather than as a measure of neuroscience literacy.

Prior to fieldwork, the 21 neuroscience-and-learning belief statements underwent a content-validation process involving five subject-matter experts in neuroscience, each with more than 10 years of academic and research experience. The experts were affiliated with the Universidad Peruana de Ciencias Aplicadas (Peru), the Universidad de Sevilla (Spain), the Universidad Nacional de Educación (Ecuador), the Universidad de Málaga (Spain), and the Universidad Nacional de Loja (Ecuador). Expert review was conducted on an item-by-item basis using a structured evaluation matrix in which each statement was examined in terms of its scientific accuracy, relevance to the construct being assessed, clarity of wording, conceptual coherence, and suitability for a university-faculty population. The review form also provided space for qualitative comments and proposed reformulations whenever an expert considered that an item required modification.

The reviewers’ observations primarily concerned the precision of neuroscientific terminology, the degree of generalization expressed in some statements, the presence of potentially ambiguous qualifiers, and the need to ensure that each item conveyed one clearly identifiable proposition. In particular, the experts recommended reducing categorical or deterministic wording in statements referring to relationships between brain functioning and learning when the available evidence supports conditional rather than universal effects. They also suggested distinguishing more clearly between claims concerning neural or cognitive processes and claims concerning their direct educational implications, since the existence of a neuroscientific mechanism does not necessarily imply a specific instructional effect. Additional recommendations addressed statements in which contextual conditions—such as population characteristics, duration, intensity, or learning task—could substantially alter the scientific interpretation of the claim.

These recommendations resulted in several types of revisions. First, expressions that could be interpreted as universal assertions were reformulated to delimit the scope of the claim and reduce unintended absolutist interpretations. Second, terminology referring to brain processes, memory, learning, stress, sleep, and neuroplasticity was standardized to improve conceptual precision across the questionnaire. Third, statements containing potentially overlapping ideas were simplified so that respondents could evaluate a single proposition rather than two related claims simultaneously. Fourth, wording that could implicitly suggest a scientifically preferred response was revised to make the statements more neutral and to reduce response bias. The purpose of these revisions was not to change the constructs being investigated but to ensure that respondents’ answers reflected their beliefs about the intended proposition rather than different interpretations of imprecise wording.

After incorporation of the expert feedback, the revised instrument was pilot-tested with 27 university faculty members. The pilot administration examined item comprehensibility, response-category functioning, possible semantic ambiguity, and the overall consistency of the belief statements before full deployment. Feedback from the pilot served to identify expressions that remained difficult to interpret and to introduce minor linguistic adjustments, with no intended change in the substantive meaning of the items. Internal consistency was subsequently assessed using Cronbach’s alpha, yielding a coefficient of 0.80 for the pilot version of the belief statements, which supported their use in the subsequent field administration. The documentation of expert review and pilot testing was recovered after the first round of revision, which is why these procedures can now be described in detail. The surviving records do not, however, permit participant-level linkage between the 27 pilot respondents and the definitive consent-filtered dataset of 269. The 269-record consent-filtered dataset is the sole dataset used for the analyses, tables and figures reported here, and the pilot is not claimed to constitute a known subset of it.

Internal consistency was examined with Cronbach’s alpha for several item sets. Bivariate comparisons of the alignment percentage used a Mann–Whitney *U* test for gender and Kruskal-Wallis tests for age, experience, collapsed academic degree, and collapsed education-related training. Information-source comparisons used Mann–Whitney *U* tests. Rank-biserial correlation was reported for two-group comparisons.

To account for repeated responses within participants, an exploratory generalized estimating equation logistic model was fitted to 4,033 non-missing item-level alignment outcomes. The model used a binomial distribution, logit link, exchangeable working correlation, participant clustering, and fixed effects for the 15 items. Covariates were gender, teaching-experience category, collapsed degree, collapsed education-related training, and indicators for formal curriculum, courses, scientific literature, social media, and artificial-intelligence tools as information sources. Adjusted odds ratios and robust 95% confidence intervals are reported. Benjamini-Hochberg false-discovery-rate adjustment was applied across the 14 participant-level covariates. Age was included in the objectives and in the bivariate analyses but was excluded from the adjusted model because its categories overlap substantially with the teaching-experience categories; retaining both would have introduced collinearity between two ordinal proxies for career stage, and teaching experience was retained as the more directly interpretable of the two. A specification replacing teaching experience with age is reported in Supplementary Table S3. Categories were collapsed to avoid sparse cells: academic degree was collapsed into below master’s (technical, bachelor’s or engineering, and specialization; *n* = 29), master’s (*n* = 188), and doctorate (*n* = 52); education-related training was collapsed into none (*n* = 46), short pedagogical courses (*n* = 138), bachelor’s degree or specialization in education (*n* = 30), and master’s or doctorate in education or an allied field (*n* = 55). The reference categories were men for gender, 0–5 years for teaching experience, below master’s for degree, none for education-related training, and not selected for each information source. The model converged without warnings and no separation or non-identifiability was observed after collapsing. Sensitivity to the assumed dependence structure was examined by refitting the model with an independence working correlation and cluster-robust standard errors. Analyses were reproduced in Python 3.13 using NumPy, SciPy, and statsmodels. The item-level dataset is openly deposited in Zenodo under the digital object identifier. The recoding rules and the item-classification dictionary accompany this submission, so that every quantity reported below may be recomputed from the data themselves, as recorded in the Data Availability Statement.

Analytical provenance. Every estimate reported in this article was computed from the corrected, consent-filtered analytical file described in the Design and Participants subsection, which holds the 269 consented records and a binary gender variable comprising 131 women and 138 men. That file is openly deposited and the analysis code that reads it accompanies this submission, so every quantity reported below can be recomputed independently.

Two earlier versions of this manuscript reported participant-level estimates computed before that correction, when the gender variable of 24 records still carried the values of the raw export; those estimates are superseded here. The consequences of the correction are circumscribed and are stated so that the two sets of figures may be reconciled. Gender is at once the grouping variable of the bivariate comparison of alignment percentages and a covariate of the adjusted model, so a change in it alters the group means, the Mann–Whitney *U* statistic and its rank-biserial correlation, every adjusted odds ratio in the model, the associated confidence intervals, and the Benjamini-Hochberg-adjusted *p*-values, which are computed across the whole family of participant-level covariates and consequently move together. All of these were recomputed on the corrected file, together with the sensitivity specifications and the forest plot that displays them. Quantities that do not involve gender are unaffected and are identical across versions: the item-level agreement proportions and Wilson intervals, the neuromyth endorsement counts, the mean alignment percentage for the sample as a whole, and the participant-level correlation bearing on the coexistence hypothesis.

### 3.4 Ethical considerations

The study adhered to the ethical principles for research involving human participants set out in the Declaration of Helsinki (World Medical Association, 2013) and to the institutional requirements governing educational research at the participating university. Ethical approval for the original survey was granted by the Consejo de Investigación—Comité de Ética of the Universidad Politécnica Salesiana under protocol number 4985817-3 in October 2025, prior to the commencement of fieldwork. The original data collection was conducted by the Grupo de Innovación Educativa Neuroeduca (GIE), which acted as data controller. That approval covered the original data collection; the present secondary reanalysis was carried out on the de-identified dataset, within the purposes disclosed in the consent statement, and required no further contact with participants. Processing complied with the applicable national data-protection legislation, and the legal basis recorded in the consent statement is the free, specific and informed consent of the data subject, given electronically before any item was presented. The survey was administered anonymously through Microsoft Forms: names, national identity numbers and email addresses were not requested, and the file used for this reanalysis contains no such fields. The consent statement further specified restricted access, anonymization of results in publications, and the erasure or irreversible anonymization of the records once the research is complete; these terms govern the availability of the dataset as described in the Data Availability Statement. The ethics information reported here corresponds exactly to that entered in the submission system.

Participation was voluntary and uncompensated. Before responding, every faculty member was presented with an informed-consent statement describing the purpose of the study, the voluntary nature of participation, the absence of foreseeable risks, the approximate time required, and the intended processing of their responses; all 269 records analyzed corresponded to respondents who affirmatively provided this consent, including consent for the described processing of personal data. Respondents were informed that they could decline to participate or withdraw at any point without consequence.

Data protection was governed by the principles of minimization, confidentiality, and purpose limitation. The instrument collected categorical survey responses and coarse demographic categories rather than direct identifiers, and the analytical dataset used for this reanalysis contained no names, contact details, institutional identifiers, or other information permitting the re-identification of individual participants. Responses were stored securely and processed in aggregate; the two missing responses documented above were retained transparently rather than imputed. Because the present work constitutes a secondary reanalysis of previously collected, de-identified data, no additional contact with participants was required, and the analysis was confined to the purposes disclosed at the point of consent.

### 3.5 Participant profile

Women represented 48.7% of participants and men 51.3%. Nearly half were 30–44 years old (48.3%), and 36.4% reported five or fewer years of teaching experience. Most participants held a master’s degree (69.9%), followed by a doctorate (19.3%). Short pedagogical courses were the most common form of education-related training (51.3%). The most frequently selected information sources were courses or professional development (61.3%) and scientific books or journals (43.5%); 26.0% selected artificial-intelligence tools. These figures describe the participating sample and should not be generalized to all Ecuadorian university faculty members without a documented probability-sampling frame. Table 1 summarizes the characteristics of the participating sample.

## 4 Results

### 4.1 Item-level endorsement

**Figure 1:**
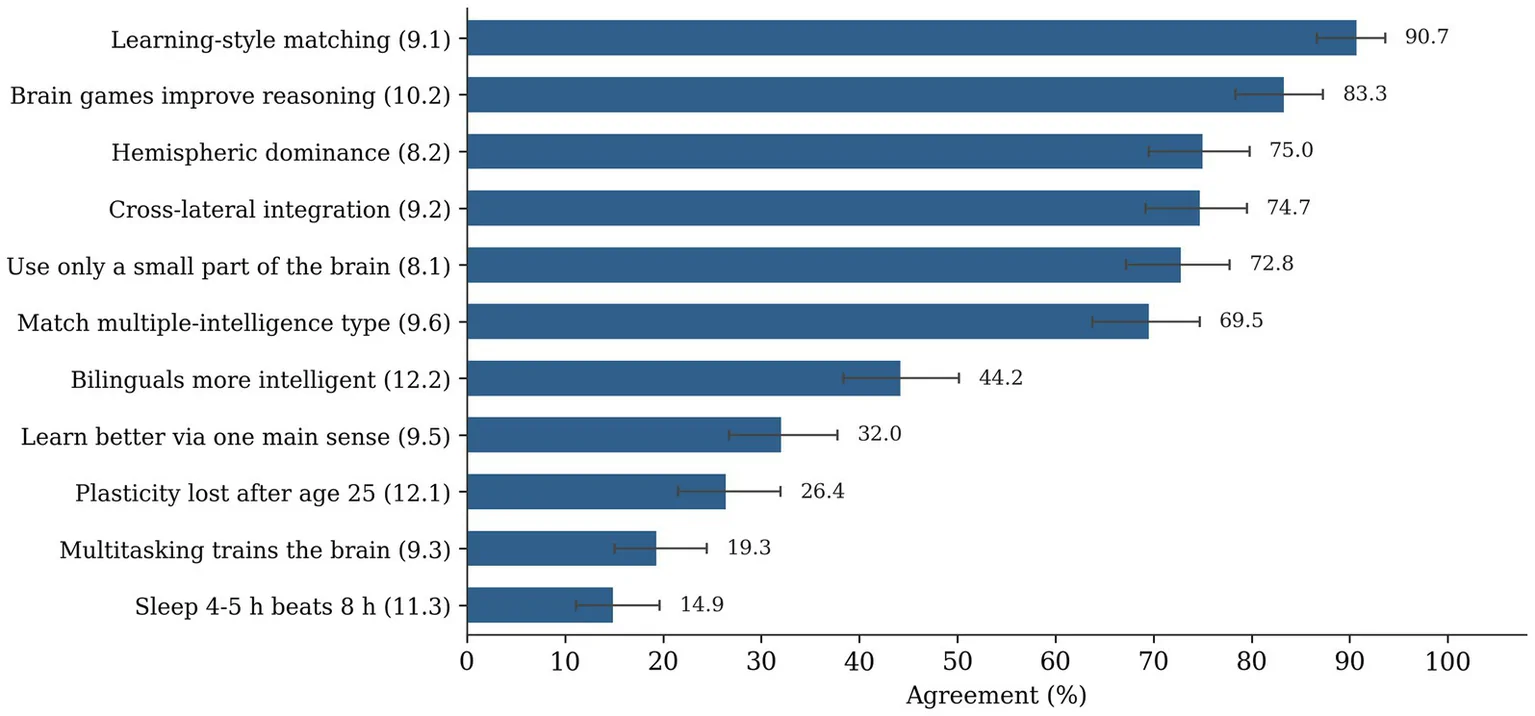
Agreement with the 11 established neuromyths or unsupported general claims. Error bars show Wilson 95% confidence intervals.

The first specific objective and hypothesis H1 concerned the prevalence of established educational neuromyths. Figure 1 presents agreement with the 11 statements classified as established neuromyths or unsupported general claims, ordered by magnitude and accompanied by Wilson 95% confidence intervals. Endorsement was highest for matching teaching to a visual, auditory, or kinesthetic learning style (90.7, 95% CI [86.7, 93.6%]), a proportion whose lower confidence bound remains well above four in five respondents and thus indicates that agreement with this claim was very widespread within the participating sample. It was followed by generalized transfer from brain games (83.3, 95% CI [78.3, 87.3%]), hemispheric dominance construed as an analytical-versus-creative classification (75.0, 95% CI [69.5, 79.8%]), cross-lateral exercises as a means of integrating the hemispheres and improving literacy (74.7, 95% CI [69.2, 79.6%]), and the belief that humans use only a small part of the brain (72.8, 95% CI [67.1, 77.7%]). For each of these five claims the entire confidence interval lies above the majority threshold of 50%, indicating that endorsement within the participating sample exceeded a split of opinion; because recruitment was non-probabilistic, these intervals quantify precision within the sample rather than prevalence among Ecuadorian university faculty at large.

Not every myth attracted majority assent, and the gradient is itself informative. Three statements fell below one third: learning better through a single main sense (32.0%), loss of plasticity after age 25 (26.4%), and the notion that multitasking trains the brain toward greater efficiency (19.3%), while agreement that 4-5 hours of sleep consolidates information better than 8 hours reached only 14.9%. This dispersion should not, however, be read as evidence of uniformly high literacy, inasmuch as the same respondents could reject one claim while endorsing several others; the analysis of coexistence below quantifies precisely this pattern. Taken together, the concentration of endorsement among the most pedagogically consequential claims—learning styles, broad brain-game transfer, and hemispheric dominance that supports H1. Full item-level results for all 21 statements, including class, agreement, neutral, and disagreement percentages, are reported in Table 2.

**Table 2:** Responses to the 21 neuroscience-and-learning statements.

Item	Abbreviated statement	Class	Agree %	Neutral %	Disagree %
8.1	Use only a small part of the brain	M/U	72.8	7.1	20.1
8.2	Analytical/creative people use a dominant hemisphere	M/U	75.0	15.3	9.7
9.1	Teaching should match visual/auditory/kinesthetic style	M/U	90.7	6.3	3.0
9.2	Cross-lateral exercises integrate hemispheres and improve literacy	M/U	74.7	21.6	3.7
9.3	Multitasking trains the brain to be more efficient	M/U	19.3	22.3	58.4
9.4	Long projects outperform frequent assessment by reducing working-memory load	C	51.3	26.4	22.3
9.5	Students learn better through one main sense	M/U	32.0	24.2	43.9
9.6	Teaching should match a predominant multiple intelligence	M/U	69.5	20.1	10.4
10.1	Classical music temporarily improves spatial reasoning	C	62.8	32.0	5.2
10.2	Brain games improve general abstract reasoning	M/U	83.3	13.0	3.7
11.1	Vitamins/omega-3 improve memory in healthy students	C	67.7	24.2	8.2
11.2	High hydration improves cognition and prevents brain fog	C	78.4	19.7	1.9
11.3	Sleeping 4–5 h consolidates better than 8 h	M/U	14.9	10.8	74.3
12.1	Brain plasticity is lost after age 25	M/U	26.4	34.6	39.0
12.2	Bilingual students have higher intelligence	M/U	44.2	30.1	25.7
12.3	Fixed morning/evening types justify personalized schedules	C	52.0	33.8	14.1
12.4	Prolonged academic stress can durably affect learning	E	82.2	9.3	8.6
12.5	Men and women differ in verbal/spatial memory	C	57.6	21.6	20.8
13.1	The brain can learn and adapt throughout life	E	91.8	4.8	3.3
13.2	Adequate sleep supports memory consolidation	E	98.1	1.5	0.4
13.3	Spaced practice supports long-term learning	E	95.5	3.3	1.1

M/U, established neuromyth or unsupported general claim; E, evidence-aligned; C, context-dependent or overgeneralized. Percentages may differ slightly from 100 because of rounding. Context-dependent items were not included in the scientific-alignment indicator.

### 4.2 Coexistence of evidence-aligned knowledge and neuromyths

The second objective and hypothesis H2 required adjudicating between two competing accounts: a deficit model, under which neuromyth endorsement reflects a simple absence of knowledge, and a coexistence model, under which accurate knowledge and misconception co-occur. Two quantities bear directly on this contrast. On the one hand, participants endorsed a mean of 6.02 of the 11 neuromyth or unsupported statements (SD = 2.42, median = 6, interquartile range = 5–7); 98.1% endorsed at least one, 75.5% at least five, and 41.6% at least seven, so that averaged across the eleven claims 54.8% of participants agreed with the neuromyth wording while only 26.5% correctly rejected it. On the other hand, and for the same respondents, agreement with the four evidence-aligned statements averaged 91.9%: 98.1% affirmed that adequate sleep contributes to memory consolidation, 95.5% that spaced practice supports long-term learning, 91.8% that the brain can learn and adapt throughout life, and 82.2% that prolonged academic stress can durably affect learning.

**Figure 2:**
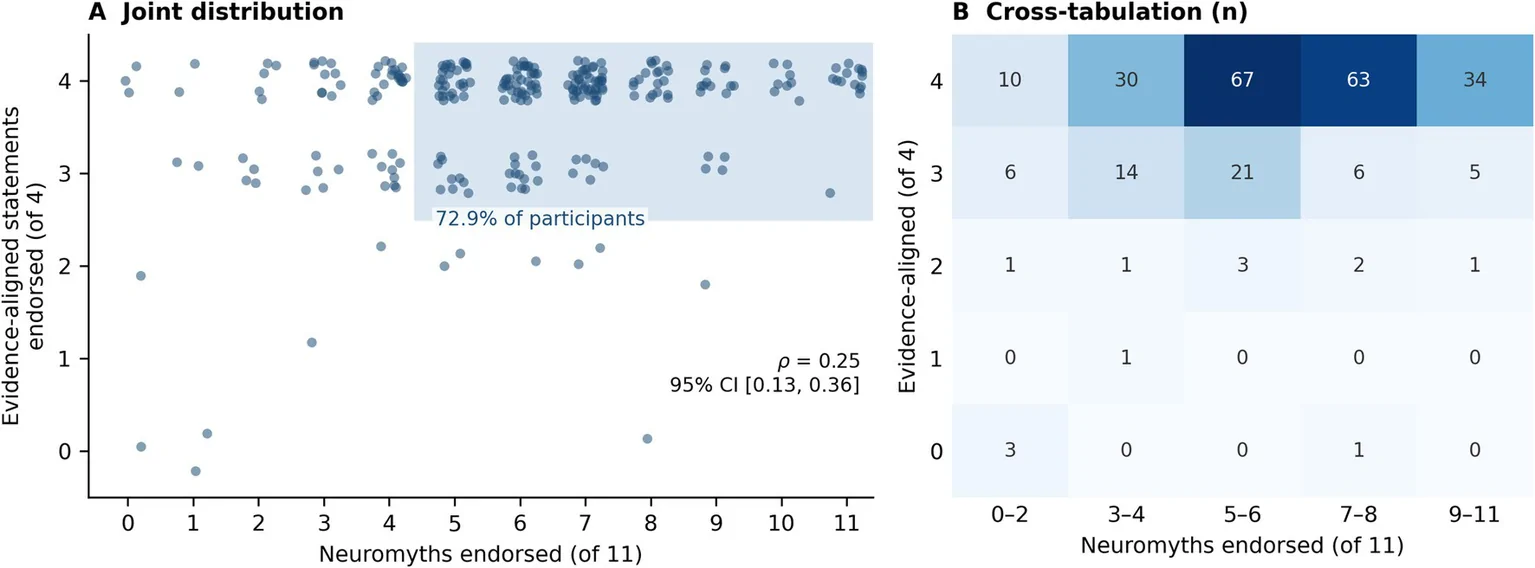
**(A)** Participant-level joint distribution of evidence-aligned knowledge and neuromyth endorsement. Each point represents one of the 269 participants, plotting the number of the four evidence-aligned statements endorsed (vertical axis, jittered) against the number of the 11 neuromyth or unsupported statements endorsed (horizontal axis, jittered). The shaded region marks participants who endorsed at least three evidence-aligned statements together with five or more neuromyths. Point size is constant; jitter is applied on both axes for legibility. **(B)** Cross-tabulation of the same two variables (cell counts).

Were the deficit model correct, high adherence to evidence-aligned principles should coincide with low neuromyth endorsement; in these data a 91.9% mean endorsement of accurate principles sits alongside a mean of six endorsed myths per respondent. Two aggregate quantities cannot, however, settle the matter: averages computed over the whole sample do not establish how evidence-aligned knowledge and neuromyth endorsement are related within individuals, and reading a within-person relationship from them would amount to an ecological inference. The question was therefore addressed directly at participant level. The association between each participant’s evidence-aligned score and the number of neuromyths endorsed was estimated with a Spearman rank correlation *ρ* = 0.25, 95% CI [0.13, 0.36], *p* < 0.001; Kendall’s *τ* = 0.21, *p* < 0.001, and the joint distribution of the two variables is displayed in Figure 2; 72.9% of participants endorsed at least three of the four evidence-aligned statements while also endorsing five or more neuromyths, and 61.0% endorsed all four evidence-aligned statements while endorsing five or more neuromyths. The observed association is weak and positive rather than negative, so the data do not support the inverse relationship a deficit account predicts; accurate knowledge and specific misconception behave as partly independent dimensions rather than as opposite ends of a single continuum. Two qualifications constrain this reading. First, the positive sign should not be interpreted as indicating that greater accurate knowledge fosters misconception: because every statement was phrased in the affirmative, an acquiescent response set inflates agreement with true and false statements alike, and agreement with the six context-dependent items, which enter neither score, correlates with neuromyth endorsement at *ρ* = 0.53 and with evidence-aligned endorsement at *ρ* = 0.24, which is the signature of such a set. Second, the evidence-aligned score is severely restricted in range (95.2% of participants endorsed at least three of its four items), so the coefficient is attenuated and estimated over a narrow band; computed on the mean Likert rating of the four evidence-aligned items rather than on the dichotomised count, the correlation is *ρ* = 0.04 (*p* = 0.56). Both specifications agree in the respect that matters for H2: there is no evidence of the negative association a deficit model requires.

### 4.3 Predictors of scientifically aligned responding

The third objective, addressing hypotheses H3 and H4, examined whether scientific alignment is conditioned by demographic and professional factors. The exploratory 15-item alignment score, whose properties frame these tests, had a mean of 44.0% (SD = 15.1), a median of 46.7%, and an interquartile range of 33.3–53.3%. Internal consistency was alpha = 0.77 for the 11 neuromyth items, 0.68 for the four evidence-aligned items, 0.72 for the 15 directionally aligned Likert items, and 0.69 for the 15 binary alignment indicators; these values suffice for exploratory group-level analysis, although the mixed constructs and uniformly positive wording caution against treating the composite as a validated unidimensional measure of neuroscience literacy, and item-level outcomes accordingly retain primacy.

Consistent with H3, no statistically robust association was detected between the broad categorical predictors and alignment. Kruskal-Wallis tests returned no significant differences by age (*H* = 4.85, *p* = 0.183), teaching experience (*H* = 3.87, *p* = 0.276), collapsed academic degree (*H* = 3.64, *p* = 0.162), or collapsed education-related training (*H* = 7.41, *p* = 0.060), the last approaching but not attaining the conventional threshold. Two associations did emerge in bivariate analysis: women exhibited a higher mean alignment percentage than men (46.7% vs. 41.4%; *U* = 10,821, *p* = 0.005, rank-biserial *r* = 0.20, a small effect), and respondents citing formal academic curricula as a source scored higher than those who did not (46.9% vs. 42.8%; *U* = 8,581, *p* = 0.021, *r* = 0.18). The remaining information sources were non-significant. These tests failed to reject the null hypothesis; they do not demonstrate that the corresponding associations are absent.

**Figure 3:**
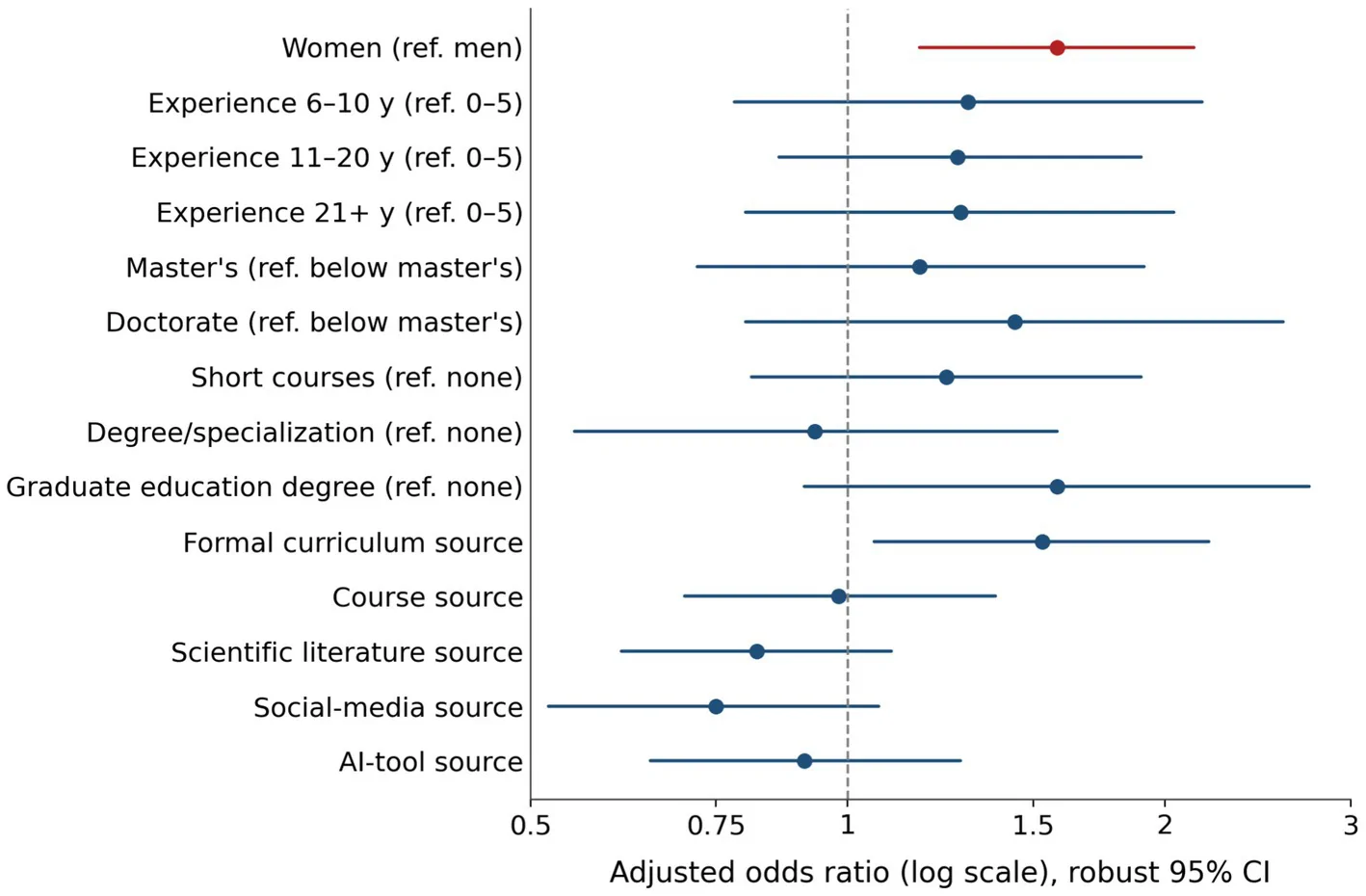
Forest plot of adjusted odds ratios from the generalized estimating equation model for an evidence-aligned item response. Points are adjusted odds ratios; horizontal lines are robust 95% confidence intervals on a logarithmic scale. The reference line is at an odds ratio of 1.0. The female-versus-male contrast (red) was the only covariate that remained significant after Benjamini-Hochberg correction. Under the primary specification, in which neutral responses count as not aligned; the contrast does not retain significance when neutral responses are excluded.

The clustered generalized estimating equation model, which accounts for the repeated item responses nested within participants, arbitrated these associations under adjustment and multiplicity control. The gender association was detected under the primary specification: women had 1.58 times the odds of an aligned response relative to men (95% CI [1.17, 2.13], nominal *p* = 0.003, Benjamini-Hochberg-adjusted *p* = 0.036), the confidence interval excluding unity. This exploratory estimate is nevertheless conditional on the dichotomisation and item-classification decisions described in the Methods, and it is not stable across them. The direction and magnitude persist under an independence working correlation (aOR = 1.55, BH-adjusted *p* = 0.075) and when age replaces teaching experience (aOR = 1.56, BH-adjusted *p* = 0.045), and the ordinal cumulative-logit specification points the same way (OR = 1.24, 95% CI [1.10, 1.40], without cluster-robust adjustment). When neutral responses are excluded rather than counted as not aligned, however, the estimate attenuates to aOR = 1.51 (95% CI [1.04, 2.19], nominal *p* = 0.029) and no longer survives false-discovery-rate correction (adjusted *p* = 0.249); mean alignment under that coding rises to 51.9%. The gender association should therefore be reported as suggestive and coding-dependent rather than as a robust effect. Supplementary Table S2 reports all specifications. The formal-curriculum association, by contrast, attenuated under correction (adjusted odds ratio = 1.53, 95% CI [1.06, 2.20], nominal *p* = 0.023, adjusted *p* = 0.159) and is therefore best regarded as a replication hypothesis rather than an established effect. No other participant-level covariate reached significance; several intervals nevertheless remain compatible with associations of practical interest, the odds ratios for the three teaching-experience categories range from 1.27 to 1.30 with upper bounds reaching 2.17, so these results indicate that no robust association was detected rather than that none exists. The estimated within-participant working correlation was 0.133; repeated responses nested within participants require an appropriate dependence structure irrespective of the magnitude of that estimate. Figure 3 arrays the adjusted odds ratios against the null of no effect, and Table 3 reports the full model. The gender finding satisfies the exploratory H4. It is descriptive rather than explanatory and bears on a question distinct from H3: that the one covariate for which an association was detected is neither degree, experience, nor training does not itself constitute evidence against a deficit model, and no such inference is drawn here.

**Table 3:** Generalized estimating equation model for an evidence-aligned item response.

Predictor (reference)	aOR	95% CI	*p*	BH *p*
**Female (men)**	**1.58**	**[1.17, 2.13]**	**0.003**	**0.036**
Experience 6–10 years (0–5)	1.30	[0.78, 2.17]	0.315	0.458
Experience 11–20 years (0–5)	1.27	[0.86, 1.90]	0.233	0.458
Experience 21+ years (0–5)	1.28	[0.80, 2.04]	0.297	0.458
Master’s degree (below master’s)	1.17	[0.72, 1.91]	0.523	0.666
Doctorate (below master’s)	1.44	[0.80, 2.59]	0.227	0.458
Short courses (none)	1.24	[0.81, 1.90]	0.327	0.458
Degree/specialization (none)	0.93	[0.55, 1.58]	0.794	0.855
Graduate education degree (none)	1.58	[0.91, 2.74]	0.101	0.408
Formal curriculum source (not selected)	1.53	[1.06, 2.20]	0.023	0.159
Course source (not selected)	0.98	[0.70, 1.38]	0.928	0.928
Scientific literature source (not selected)	0.82	[0.61, 1.10]	0.186	0.458
Social-media source (not selected)	0.75	[0.52, 1.07]	0.117	0.408
AI-tool source (not selected)	0.91	[0.65, 1.28]	0.604	0.705

aOR, adjusted odds ratio; CI, robust confidence interval; BH *p*, Benjamini-Hochberg false-discovery-rate-adjusted *p*-value. The model also included fixed effects for the 15 scored items. Odds ratios describe association, not causation. All estimates in this table were computed from the consent-filtered analytical file described in the Data Analysis subsection, which is the definitive dataset for every result reported in this article. Bold values indicate the covariate that remained statistically significant after Benjamini–Hochberg false-discovery-rate correction (BH *p* < 0.05).

Read jointly, the three sets of analyses support the following reading. H1 is supported by the widespread endorsement of the most consequential myths within this sample; the evidence bearing on H2 rests on the participant-level analysis reported above rather than on the aggregate profiles alone; with respect to H3, no statistically robust association was detected for degree, experience, or training, which is consistent with the hypothesis but does not by itself establish that such associations are absent; and the exploratory H4 is satisfied by a single gender association that awaits replication. An affirmative claim of negligible effects for the H3 predictors would require an equivalence-testing or Bayesian framework with a pre-specified smallest effect size of interest, which the present secondary design does not support.

## 5 Discussion

### 5.1 Principal findings

Neuromyths coexisted with robust endorsement of accurate learning principles. Nearly all participants acknowledged the importance of adequate sleep, spaced practice, and lifelong plasticity, and yet the average participant endorsed six of eleven neuromyth or unsupported statements. This configuration supports item-specific analysis, inasmuch as a single factual-knowledge score can obscure misconceptions of direct instructional relevance (Grospietsch and Mayer, 2019; Macdonald et al., 2017). The pattern converges strikingly with von Hausen et al. (2026), who report an analogous cross-domain gradient among Chilean educators in which educational neuromyths yield the highest proportion of incorrect responses while general brain knowledge yields the fewest, corroborating the contention that neuroscience literacy is multidimensional rather than monolithic.

Learning-style matching commanded the assent of more than nine in ten participants. Multiple representations may well support learning, but they neither validate fixed learner categories nor license a general matching rule. The modest pooled benefit reported by Clinton-Lisell and Litzinger (2024), coupled with limited crossover evidence and methodological heterogeneity, affords no warrant for enshrining style diagnosis as institutional policy.

The elevated endorsement of brain-game transfer, hemispheric dominance, and cross-lateral integration bespeaks the extension of genuine phenomena beyond their demonstrated outcomes. Task practice, relative specialization, and coordinated movement do not establish far transfer to reasoning, learner taxonomies, or literacy. Educational interpretation must therefore preserve the boundary separating neural plausibility, task-specific change, and meaningful transfer (Howard-Jones, 2014; Simons et al., 2016).

### 5.2 Why more education did not guarantee better alignment

The original narrative anticipated sharper recognition with higher degrees and weaker recognition with greater experience. The reanalysis substantiated neither expectation: degree and experience proved non-significant in both bivariate and adjusted analyses. Advanced disciplinary study may omit the science of learning altogether, while experience can subsist alongside familiar yet unexamined beliefs. The result does not imply that education or experience is inconsequential; it indicates, more precisely, that no robust association between these broad categories and alignment was detected in the present sample, a conclusion consonant with von Hausen et al. (2026), for whom neither objective brain knowledge nor self-perceived expertise conferred general protection against endorsement.

Education-related training approached, without attaining, the conventional bivariate threshold. Categories such as short courses aggregate programs of markedly divergent content and intensity. Because targeted science-of-learning instruction can demonstrably reduce endorsement (Ferreira and Rodríguez, 2022), future surveys ought to measure duration, recency, provider, curriculum, assessment, and explicit refutation.

Formal curriculum exhibited a nominal association that did not survive multiplicity correction and should accordingly be entertained as a replication hypothesis. The selection of scientific books or journals was likewise non-significant; the source question, however, indexed reported exposure rather than quality, frequency, comprehension, or critical appraisal.

### 5.3 Interpreting the gender association

The gender association, which satisfies the exploratory H4, merits interpretive restraint precisely because it was the sole covariate for which an association was detected. Women exhibited higher odds of an aligned response than men, and the association withstood false-discovery-rate adjustment under the primary specification, though not when neutral responses were excluded from the outcome; it should nonetheless not be reified as an inherent gender difference. The dataset omits disciplinary field, institution, professional role, research-methods preparation, and the content or recency of training, each a plausible confound, and the result is accordingly descriptive, context-specific, and in need of replication. The association also depends on the dichotomisation of the outcome and on the item classification, and it is therefore reported alongside the sensitivity analyses in Supplementary Table S2; it persists in direction across those specifications but not in statistical significance, and is accordingly reported as suggestive and coding-dependent rather than as an established effect. Whether alignment differs by gender and whether a deficit model accounts for neuromyth endorsement are, moreover, analytically distinct questions, and no support for a conclusion about the second is drawn from the first.

Group distributions overlap substantially, moreover, and many psychological gender differences are small or highly context-sensitive (Hyde, 2005). Professional learning should therefore be calibrated to demonstrated item-level needs rather than to demographic labels, an inference that also guards against over-reading H4 beyond its exploratory status.

### 5.4 Implications for faculty development

The coexistence pattern favors targeted refutation over a general lecture on brain anatomy. A useful sequence is to state the claim, identify its kernel of truth and unsupported inference, present the strongest corrective evidence with limitations, and replace the claim with an actionable alternative. For learning styles, alternatives include content-appropriate multiple representations, attention to prior knowledge, spacing, retrieval, and feedback.

Training should explicitly distinguish improvement on a practiced task, near transfer, and far transfer. Applied to brain games or coordination exercises, educators can ask what was trained, what was measured, whether an active comparison was used, how long the effect lasted, and whether it generalized.

Source-evaluation activities can compare a peer-reviewed study’s population, design, and outcomes with a commercial or social-media claim on the same topic. Because corrections can fade, programs should include delayed reassessment and application in lesson planning (Chan and Albarracín, 2023; Rousseau, 2021). Institutional systems should avoid fixed learner-type labels and prioritize the most prevalent claims identified here: learning styles, broad brain-game transfer, hemispheric dominance, cross-lateral integration, and the 10% claim.

### 5.5 Limitations

Several limitations constrain interpretation. First, the recruitment procedure, participating institutions, sampling frame, and response rate were not documented in the files available for this study. The sample therefore cannot be assumed to represent all university faculty members in Ecuador, no response rate can be reported, and no prospective sample-size or precision justification was established. Second, the design was cross-sectional and self-reported. It measures agreement with statements, not actual classroom practices, instructional spending, or student outcomes, and it cannot establish persistence over time.

Third, all belief statements were positively phrased, increasing the risk of acquiescence. Some items combine more than one claim or use imprecise qualifiers, which motivated the context-dependent category but also limits comparability with established instruments. The three-category classification was evidence-informed but was not independently coded by multiple raters; future work should use a preregistered classification and report inter-rater agreement.

Fourth, the alignment indicator is exploratory rather than a validated scale. The belief statements themselves underwent expert content validation and pilot testing before fieldwork, as documented in the Methods; what remains outstanding is not that process but an independent psychometric validation of the composite, evidence of dimensionality, temporal stability, and measurement invariance across subgroups, which could not be sought for an indicator constructed for this reanalysis rather than for the original administration. Its reliability was moderate, and the 15 items span different scientific and educational domains. Item-level estimates should therefore receive more weight than the composite. Fifth, multiple demographic and source associations were explored. False-discovery-rate adjustment reduced the risk of chance findings, but the gender association still requires independent replication. Sixth, the alignment outcome treats neutral responses as not aligned; although sensitivity analyses under alternative codings are reported, the primary estimates remain conditional on that decision. Finally, one response was missing for each of the first two items; the amount of missingness was negligible but is reported for transparency.

## 6 Conclusion

Three axes organized this inquiry, and the evidence permits the following provisional response to each. Regarding the first, educational neuromyths proved widespread among the 269 participating university faculty members in Ecuador, most conspicuously the convictions that instruction ought to be matched to visual, auditory, or kinesthetic learning styles, that brain games yield broad gains in reasoning, and that individuals may be classified by hemispheric dominance. Regarding the second, these misconceptions coexisted with firm endorsement of evidence-aligned principles concerning sleep, spacing, stress, and lifelong plasticity; the central inference is accordingly not that faculty members are bereft of neuroscience knowledge, but that accurate knowledge and specific misconception subsist together. Regarding the third, academic degree, teaching experience, and broad categories of professional training showed no robust association with scientifically aligned responses, which indicates that no such association was detected in this sample rather than that none exists; and although a gender association surfaced in exploratory analysis, it warrants interpretation as context-specific and noncausal, depends on the treatment of neutral responses, and awaits independent replication before being treated as an established effect.

From these findings a clear programmatic implication follows. Faculty development should privilege explicit, item-level refutation over generic exposition of brain anatomy: identifying the kernel of truth, explicating the unsupported inference, marshaling the pertinent evidence, and substituting a usable instructional alternative for the discredited claim. Programs should further cultivate the capacity to distinguish task practice from transfer, neural correlates from educational effects, and mere exposure to scientific information from its critical appraisal. Because the survey was cross-sectional and the records documenting recruitment could not be recovered, the findings should not be generalized to the national population; the instrument, for its part, was content-validated by external experts and pilot-tested before fieldwork, and it is the psychometric validation of the composite indicator, not that prior process, that awaits independent corroboration. It is thus incumbent upon future inquiry to mount a multi-institutional study equipped with a documented sampling frame, independently validated items, direct observation of instructional practice, and delayed post-training measurement, which together would furnish a firmer foundation for educational policy and professional development.

## Data Availability

The corrected, consent-filtered analytical dataset used in this study (*N* = 269) is openly available in Zenodo at https://doi.org/10.5281/zenodo.21998158. The recoding rules, item-classification dictionary, and analysis materials accompanying the study allow the reported analyses to be reproduced. Further inquiries can be directed to the corresponding author.
