# Supplementary material for: Educational neuromyths in higher education: beliefs about the brain and learning among university faculty members
Source: Front Psychol. 2026 Sep 9;17:1952466. doi: 10.3389/fpsyg.2026.1952466 (PMC13597814; doi:10.3389/fpsyg.2026.1952466)
Supplement: Supplementary file 1 [file Supplementary_file_1.docx]

**Supplementary Table S1. Classification of the 21 belief statements.**

| **Item** | **Statement (original Spanish / English translation)** | **Evidentiary rationale and key source** |
| --- | --- | --- |
| 8.1 | Los seres humanos utilizamos solo una pequeña parte de la capacidad total de nuestro cerebro. / Human beings use only a small part of the total capacity of the brain. | Functional imaging shows activity across the whole brain over time; the ten-per-cent claim has no empirical basis (Dekker et al., 2012; Howard-Jones, 2014). |
| 8.2 | Las personas pueden clasificarse principalmente como más analíticas o más creativas según el hemisferio cerebral que utilizan con mayor frecuencia. / People can be classified mainly as analytical or creative according to the hemisphere they use more often. | Lateralisation is task-specific and does not yield analytical or creative person types (Howard-Jones, 2014). |
| 9.1 | Los estudiantes aprenden mejor cuando la enseñanza se adapta a su estilo de aprendizaje predominante (visual, auditivo o kinestésico). / Students learn better when teaching is matched to their predominant learning style (visual, auditory or kinaesthetic). | The matching hypothesis fails when tested with crossover designs (Pashler et al., 2008; Clinton-Lisell and Litzinger, 2024). |
| 9.2 | Hacer ejercicios de coordinación motriz (como cruzar la línea media del cuerpo) ayuda a integrar los hemisferios y mejora habilidades como la lectura y escritura. / Motor-coordination exercises such as crossing the body midline integrate the hemispheres and improve reading and writing. | Midline-crossing exercises have no demonstrated effect on hemispheric integration or literacy (Dekker et al., 2012). |
| 9.3 | Realizar varias tareas al mismo tiempo, como estudiar mientras se revisa el teléfono móvil, entrena el cerebro para ser más eficiente en entornos reales profesionales. / Doing several tasks at once, such as studying while checking a mobile phone, trains the brain to be more efficient in real professional settings. | Heavy media multitasking is associated with poorer, not better, cognitive control (Ophir et al., 2009). |
| 9.4 | El aprendizaje basado en proyectos prolongados es más efectivo que las evaluaciones frecuentes porque reduce la sobrecarga de la memoria de trabajo. / Learning based on long projects is more effective than frequent assessment because it reduces working-memory overload. | Project length and assessment frequency interact with task and context; distributed retrieval practice generally aids retention (Cepeda et al., 2006). |
| 9.5 | Los estudiantes aprenden mejor cuando la enseñanza se dirige principalmente a un solo sentido (por ejemplo, visual o auditivo). / Students learn better when teaching addresses mainly a single sense (for example, visual or auditory). | Single-sense instruction is not supported; multimodal presentation is generally at least as effective (Pashler et al., 2008). |
| 9.6 | Cada estudiante posee un tipo de inteligencia predominante, por lo que la enseñanza debe adaptarse a ese tipo de inteligencia. / Each student has a predominant type of intelligence, so teaching should be matched to that type. | Multiple-intelligence typologies lack psychometric support as a basis for matched instruction (Dekker et al., 2012; Ferrero et al., 2016). |
| 10.1 | Escuchar música clásica durante el estudio intenso mejora temporalmente ciertas habilidades cognitivas, como el razonamiento espacial. / Listening to classical music during intensive study temporarily improves certain cognitive abilities, such as spatial reasoning. | Short-lived arousal effects have been reported, but not a durable cognitive gain; the claim is conditional rather than false (Howard-Jones, 2014). |
| 10.2 | El uso frecuente de juegos mentales (tipo sudokus) o aplicaciones de entrenamiento cerebral mejoran significativamente el razonamiento abstracto en distintas áreas académicas. / Frequent use of mental games (such as sudoku) or brain-training applications significantly improves abstract reasoning across academic areas. | Brain-training gains are largely task-specific and do not transfer broadly (Simons et al., 2016). |
| 11.1 | El consumo de vitaminas o suplementos nutricionales como omega-3 potencia la memoria y mejora la concentración en estudiantes universitarios sanos, en periodos de exámenes. / Vitamins or nutritional supplements such as omega-3 enhance memory and concentration in healthy university students during examination periods. | Supplementation effects depend on baseline status; benefits in already well-nourished students are not established (Howard-Jones, 2014). |
| 11.2 | Mantener una hidratación elevada durante clases prolongadas, debates o seminarios mejora el rendimiento cognitivo y previene la “niebla mental”. / High hydration during long classes, debates or seminars improves cognitive performance and prevents “brain fog”. | Dehydration impairs performance, but this does not license a universal prescription of high intake (Masento et al., 2014). |
| 11.3 | Dormir solo 4-5 horas antes de un examen final “consolida” mejor la información que dormir 8 horas completas. / Sleeping only four to five hours before a final examination consolidates information better than sleeping a full eight hours. | Sleep restriction impairs consolidation rather than aiding it (Rasch and Born, 2013). |
| 12.1 | El cerebro de los jóvenes universitarios pierde plasticidad después de los 25 años, haciendo más difícil aprender materias complejas como cálculo avanzado. / The brain of young university students loses plasticity after the age of 25, making complex subjects such as advanced calculus harder to learn. | Plasticity persists well beyond age 25, though its form changes (Magee and Grienberger, 2020). |
| 12.2 | Los estudiantes bilingües en edad universitaria tienen niveles más altos de inteligencia que los estudiantes monolingües. / Bilingual university-age students have higher levels of intelligence than monolingual students. | Bilingualism is not associated with higher general intelligence; executive-function advantages are small and contested (Lehtonen et al., 2018). |
| 12.3 | Hay estudiantes “mañaneros” o “nocturnos” cuyo cerebro aprende mejor en horarios fijos, justificando horarios personalizados en carreras. / There are “morning” and “evening” students whose brains learn better at fixed times, which justifies personalised timetables in degree programmes. | Chronotype differences are real but do not justify fixed individualised timetables (Adan et al., 2012). |
| 12.4 | El estrés académico prolongado puede afectar de forma duradera la capacidad de aprendizaje. / Prolonged academic stress can durably affect the capacity to learn. | Prolonged stress has documented effects on hippocampal and prefrontal function relevant to learning (McEwen and Morrison, 2013). |
| 12.5 | Existen diferencias cognitivas entre hombres y mujeres en habilidades como la memoria verbal y la memoria espacial. / Cognitive differences exist between men and women in abilities such as verbal and spatial memory. | Average group differences are small and overlapping and do not justify deterministic grouping (Hyde, 2005). |
| 13.1 | El cerebro mantiene la capacidad de aprender y adaptarse a lo largo de toda la vida. / The brain retains the capacity to learn and adapt throughout life. | Lifelong plasticity is well established (Magee and Grienberger, 2020). |
| 13.2 | El sueño adecuado contribuye a la consolidación de la memoria. / Adequate sleep contributes to memory consolidation. | Sleep-dependent memory consolidation is well established (Rasch and Born, 2013). |
| 13.3 | La práctica distribuida en el tiempo favorece el aprendizaje a largo plazo. / Practice distributed over time favours long-term learning. | The spacing effect is among the most replicated findings in learning research (Cepeda et al., 2006). |

*M/U = established neuromyth or unsupported general claim; E = evidence-aligned; C = context-dependent or overgeneralized. The 11 M/U and 4 E statements constitute the 15-item alignment indicator; the 6 C statements were not scored dichotomously. Classification was established on evidentiary grounds before the response distributions were inspected.*
