# Supplementary material for: Educational neuromyths in higher education: beliefs about the brain and learning among university faculty members
Source: Front Psychol. 2026 Sep 9;17:1952466. doi: 10.3389/fpsyg.2026.1952466 (PMC13597814; doi:10.3389/fpsyg.2026.1952466)
Supplement: Supplementary file 2 [file Supplementary_file_2.docx]

**Supplementary Table S2. Gender association under alternative specifications.**

| **Specification** | **Female aOR [95% CI]** | **p** | **BH p** | **Note** |
| --- | --- | --- | --- | --- |
| Primary specification (exchangeable working correlation; neutral counted as not aligned) | 1.58 [1.17, 2.13] | .003 | .036 | 4,033 item-level observations; estimated within-participant correlation .133 |
| Independence working correlation with cluster-robust standard errors | 1.55 [1.14, 2.10] | .005 | .075 | Same observations; dependence structure not modelled |
| Neutral responses excluded and treated as missing | 1.51 [1.04, 2.19] | .029 | .249 | 3,430 item-level observations; mean alignment rises to 51.9% |
| Ordinal cumulative-logit model retaining all five Likert categories | 1.24 [1.10, 1.40] | &lt;.001 | — | Item-level model without cluster-robust adjustment; all five categories retained |
| Age in place of teaching experience (full model in Supplementary Table S3) | 1.56 [1.16, 2.09] | .003 | .045 | Age collapsed to 44 years or under, 45–59, and 60 or over to avoid a sparse reference cell |

*aOR = adjusted odds ratio of an evidence-aligned item response for women relative to men, adjusted for the covariates listed in Table 3. BH p = Benjamini-Hochberg false-discovery-rate-adjusted p value across the 14 participant-level covariates; the ordinal specification was not part of that family and is reported unadjusted. The association is directionally stable across specifications but does not retain significance after correction when neutral responses are excluded.*

**Supplementary Table S3. Generalized estimating equation model with age in place of teaching experience.**

| **Predictor (reference)** | **aOR** | **95% CI** | **p** | **BH p** |
| --- | --- | --- | --- | --- |
| Female (men) | 1.56 | [1.16, 2.09] | .003 | .045 |
| Age 45–59 (44 or under) | 1.06 | [0.75, 1.49] | .749 | .885 |
| Age 60 or over (44 or under) | 0.53 | [0.29, 0.96] | .035 | .117 |
| Master's degree (below master's) | 1.28 | [0.80, 2.04] | .309 | .446 |
| Doctorate (below master's) | 1.88 | [1.04, 3.39] | .036 | .117 |
| Short courses (none) | 1.31 | [0.86, 2.01] | .210 | .340 |
| Degree/specialization (none) | 1.01 | [0.61, 1.70] | .955 | .955 |
| Graduate education degree (none) | 2.08 | [1.20, 3.60] | .009 | .057 |
| Formal curriculum source (not selected) | 1.46 | [0.99, 2.14] | .056 | .145 |
| Course source (not selected) | 0.96 | [0.68, 1.37] | .829 | .898 |
| Scientific literature source (not selected) | 0.76 | [0.56, 1.03] | .080 | .173 |
| Social-media source (not selected) | 0.77 | [0.53, 1.11] | .163 | .303 |
| AI-tool source (not selected) | 0.86 | [0.60, 1.23] | .407 | .529 |

*Model fitted to the same 4,033 item-level observations with an exchangeable working correlation, participant clustering and fixed effects for the 15 scored items. Age replaces teaching experience because the two overlap substantially as ordinal proxies for career stage; the two youngest age categories were combined because the 18–29 category (n = 15) is too sparse to serve as a reference cell.*
